# Monitoring of Carbon Fiber-Reinforced Old Timber Beams via Strain and Multiresonant Acoustic Emission Sensors

**DOI:** 10.3390/s18041224

**Published:** 2018-04-17

**Authors:** Francisco J. Rescalvo, Ignacio Valverde-Palacios, Elisabet Suarez, Andrés Roldán, Antolino Gallego

**Affiliations:** 1 Department of Applied Physics, University of Granada, Campus Fuentenueva s/n, 18071 Granada, Spain; rescalvo@ugr.es (F.J.R.); antolino@ugr.es (A.G.); 2 Department of Building Constructions, University of Granada, Campus Fuentenueva s/n, 18071 Granada, Spain; nachoval@ugr.es; 3 Department of Electronics and Computer Technology, University of Granada, Campus Fuentenueva s/n, 18071 Granada, Spain; amroldan@ugr.es

**Keywords:** timber, carbon fiber composites, acoustic emission sensors, structural monitoring

## Abstract

This paper proposes the monitoring of old timber beams with natural defects (knots, grain deviations, fissures and wanes), reinforced using carbon composite materials (CFRP). Reinforcement consisted of the combination of a CFRP laminate strip and a carbon fabric discontinuously wrapping the timber element. Monitoring considered the use and comparison of two types of sensors: strain gauges and multi-resonant acoustic emission (AE) sensors. Results demonstrate that: (1) the mechanical behavior of the beams can be considerably improved by means of the use of CFRP (160% in bending load capacity and 90% in stiffness); (2) Acoustic emission sensors provide comparable information to strain gauges. This fact points to the great potential of AE techniques for in-service damage assessment in real wood structures.

## 1. Introduction

In Europe, after decades of concrete culture, wood is witnessing a relative boom for the construction of houses and unique buildings. One relevant cause is the Energy Performance of Buildings Directive (EPBD) [1], which will require minimal energy consumption in building practices by the year 2020. Construction with wood means a roughly 50% reduction in the duration of work execution and an 80% increase in energy efficiency. In addition, less energy is required to obtain wood than other materials (basically, steel and concrete).

Typically, wood has been used in combination with other materials in several engineering sectors. Hybrid wood/carbon/glass structures can be less costly to manufacture, and there are structural (tenacity and low density) and environmental benefits to be derived from the use of renewable materials such as wood [2]. For construction purposes, wood has traditionally been combined with metal elements, whereas its combination with carbon or glass composites is still unusual. Nevertheless, many technological developments highlight the enormous expectation amid the construction sector regarding the use of composite materials.

From the structural point of view, wood is a very efficient material due to its good resistance and limited density [3]. However, structural elements are not free of numerous defects, since wood is a biological material. Moreover, meteorological factors affect in a considerable way to its durability. It can lead to a need for replacement, repair or reinforcement of some structural wood elements. In particular, repairing and reinforcement are very interesting and recommendable options in terms of saving money and time, and respecting the historical value of the element. 

**Figure 1:**
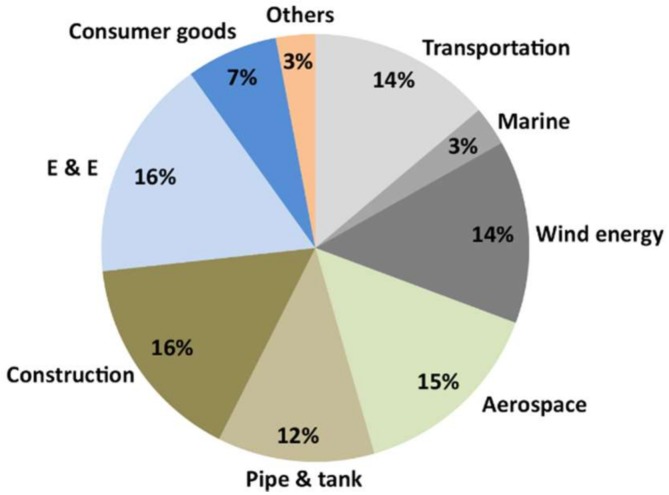
Global market of composite materials in 2017. Values in $ mil. [4].

Most traditional reinforcement systems are made with metallic components (basically steel). However, the use of composite materials for reinforcement, in particular, fiber-reinforced plastics (FRP), offers numerous advantages, such as lower extra weight and easier installation. Their development has been very important at a global level during the last decades. It is estimated that the market for composites will grow from 69.50 billion dollars in 2015 to 105.26 billion dollars in 2021. These lightweight materials are capable of withstanding high mechanical stresses, so that structures are very resistant and very light at the same time; and they have properties ensuring exceptional performance, such as corrosion resistance or high strength. Currently, the proportion of composite materials in the global construction sector amounts to 16% of the market, as seen in Figure 1 [4].

Reinforcing wood beams using FRP materials started in the 1960s [5,6,7], with GFRP (fiberglass) being proposed to reinforce sawn and glued laminated beams (glulam). Since that date, many papers have been published (see [8] for a large description of previous results and literature). However, in many previous in situ interventions, the reinforcement of timber structures of historical and non-historical building was carried out without numerical or analytical codes, i.e., following the empirical experience of the manufacturers involved and the data sheets of the FRP suppliers. For that reason, inspection methods are welcome to verify medium- and long-term integrity and functionality. Quantitative determinations of the level of damage in a structure after years in service is of foremost importance when assessing whether the activity of a building must be interrupted for eventual repair works. The importance of condition monitoring wood structures in service with monitoring sensors, like any other kind of structures, is widely acknowledged.

One of the emerging methods for monitoring damage in real time in materials such as carbon fiber-reinforced concrete is the so-called electrical resistance method [9,10]. This method is based on the breakdown of percolation network when materials are damaged, resulting in an increase in electrical resistance. These works demonstrate a good correlation between the relative change of resistance and load-deflection curves under three-point bending. Future research needs to be done in order to evaluate its effectiveness in the case of carbon fiber-reinforced wood, and also to compare with and validate the measurements of AE and strain sensors.

One of the most relevant technologies used for real-time diagnosis of structures (SHM) in nondestructive conditions consists of the use of piezoelectric sensors under the acoustic emission technique (AE) [11,12,13,14,15,16,17,18,19,20]. 

**Figure 2:**
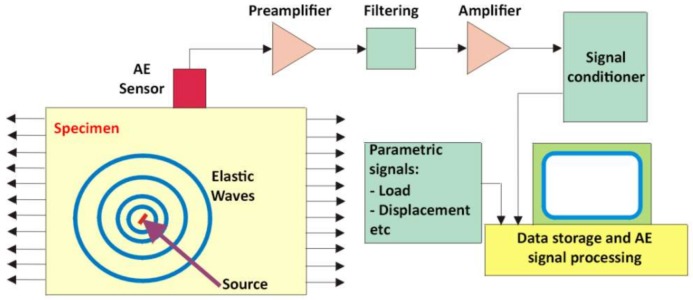
Acoustic Emission method.

Acoustic emissions (AE) are the stress waves generated by the sudden internal stress redistribution in materials or structures when changes in their strain field are produced by crack initiation and growth, crack opening and closure, deformation, dislocation movement, void formation, interfacial failure, corrosion, fiber-matrix debonding in composites, etc. These waves propagate through the material and eventually reach the surface, producing small temporary surface displacements. Usually, the stress waves are of low amplitude and of high frequency, in the ultrasonic range. This is the reason why very sensitive piezoelectric sensors are required to capture them. Due to the low amplitude of AE waves, several steps must be sequentially incorporated after their capture and before subsequent recording and analysis (Figure 2). A preamplifier is necessary to minimize the interference and prevent signal loss, while a filter is used to remove the undesirable noise and fix the frequency range of interest.

AE sources are commonly associated with damage to the material. Thus, AE detection and analysis can be used to evaluate and predict its failure [12,13,14,16,17]. The main difference between AE sensing and other non-destructive methods is the capability to inspect in real time, since continuous monitoring in situ can be carried out, and the onset and progression of damage to a structure can be assessed, even when this is not possible by means of visual inspection. Some applications of the AE method to the study of wood behavior can be found in [21,22,23,24,25,26], dealing with detection of failure in adhesive joints and evaluation of fractures and its propagation in wood.

In this work, bending tests on timber beams were monitored with multi-resonant AE sensors to demonstrate the promising use of these sensors and technique for damage monitoring in real applications. Moreover, in order to validate the monitoring process, a comparison is shown between the measurements obtained by AE sensors and traditional strain sensors. 

Furthermore, the present paper uses a layout proposed by our research group for reinforcing timber beams with carbon composite materials (CFRP), tentatively named Braided Reinforcement. It consists of the combination of a laminate strip attached on the tension side and a CFRP fabric discontinuously wrapping the timber element. The experimental study involved small and large timber beams of *Pinus Sylvestris* L. with many defects [8,24]. A comparison between non-reinforced and Braided reinforced (with unidirectional and bidirectional fabric) small-sized specimens (1.3 m long) was carried out. Results demonstrate that the bending load capacity can be improved, to the same degree as the traditional U-shaped layout with respect to non-reinforced beams. The main difference is the 50% reduction in the material used, which is very important for economic and practical reasons. Afterwards, a comparison of large-sized beams (4.5 m long) was carried out, in order to verify the improvements of this layout in terms of bending load capacity, stiffness, maximum deflection and ductility in real-sized elements. Results of accumulative AE energy released by the specimen during the test and its location are compared with the visual observation of cracks and with the measurements carried out with strain sensors.

## 2. Specimens and Mechanical Test Description

The wood beams were of *Pinus Sylvestris* from southern Spain, in service for 200–300 years. Two sizes of beams were involved in this study: (i) small beams with a cross-section of (75 ± 1.5) × (140 ± 1) mm^2^ and a length of 1288 ± 2 mm; (ii) large beams with a cross-section of (147 ± 11) × (222 ± 6) mm^2^ and a length of 4500 ± 2.4 mm. Specimen nomenclature and descriptions are summarized in Table 1 for all beams, small and large.

All the reinforcement materials were provided by the DRIZORO^®^ firm (Madrid, Spain). In particular, the CFRP pultruded laminate COMPOSITE 1405 and 1410, and the CFRP fabrics DRIZORO^®^ WRAP 200 (unidirectional) and DRIZORO^®^ CARBOMESH 210 (bidirectional) were used for all experimental works. Epoxy resin RM-CS manufactured by DRIZORO^®^ S.A.U was used as adhesive between the wood and the CFRP reinforcement. More details can be found in Rescalvo et al. [8] and Rescalvo et al. [27].

**Figure 3:**
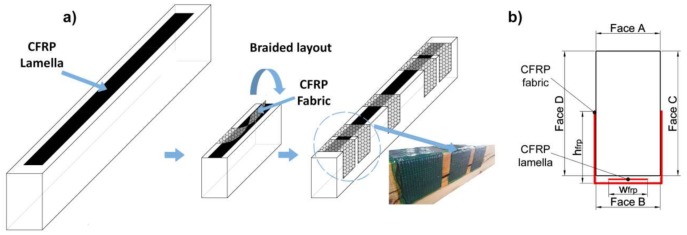
(**a**) Graphic description of the fabric wrapping process for the braided reinforcement layout. (**b**) Cross-sectional view of the braided reinforcement layout.

The reinforcement of all specimens was made by means of CFRP pultruded laminate (CFRP lamella) on the bottom side of the beam (see Figure 3b). The laminate was applied using w_frp_ = 45 mm and w_frp_ = 100 mm for small- and large-sized beams, respectively. h_frp_ was set at 75 mm in all cases. Moreover, a CFRP fabric was placed discontinuously wrapping the CFRP laminate, as shown in Figure 3a. This reinforcement layout was tentatively called Braided Reinforcement. For the small beams (1300 mm long), unidirectional and bidirectional CFRP fabrics were considered, for later comparison [8,27]. In the case of the large specimens (4500 mm long), only CFRP bidirectional fabric was used. 

**Figure 4:**
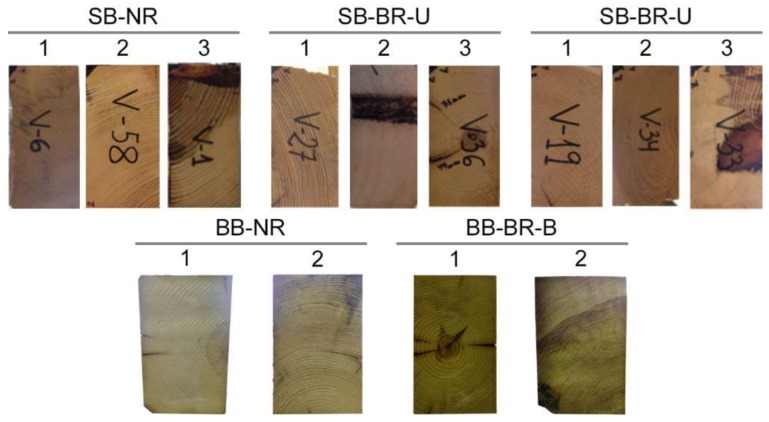
Images of the cross-section of the growth rings of the tested beams.

**Figure 5:**
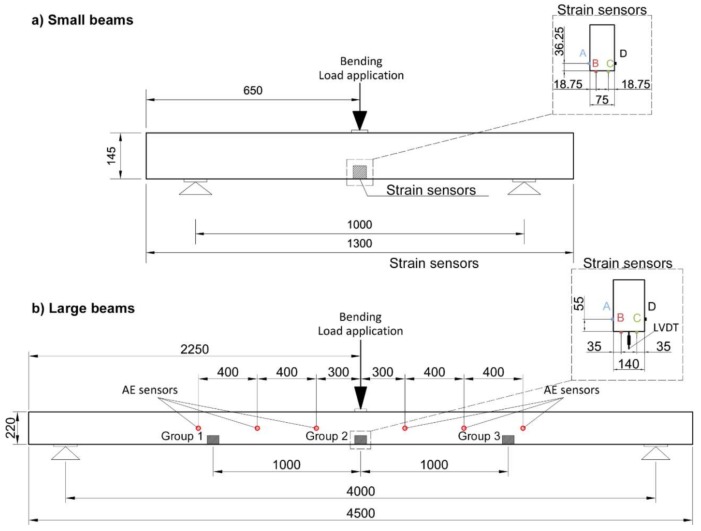
Experimental setup for small beams (**a**) and large beams (**b**). Distances in mm.

Figure 4 shows an image of the cross-section of one of the extremes of the tested beams where the distribution of the growth rings can be observed. The bottom face of each image corresponds to the tensile stressed face (i.e., the bottom face) during the bending test. All the timber beams were loaded under 3-point bending monotonic tests until failure occurred, controlling the displacement rate to 1.5 mm/min (see Figure 5).

## 3. Monitoring Sensors

### 3.1. Strain Sensors

The strains were measured using several groups of four strain sensors each. Two of them (strain sensors B and C) were put on the bottom face, while the other two (strain sensors A and D) were placed on each lateral face. Measurements were recorded with the HBM QUANTUMX MX 1615B equipment. For small beams, only one group of four strain sensors was installed on the center of the beams, while for large beams, three groups were used, as shown in Figure 5.

### 3.2. Multi-Resonant Acoustic Emission Sensors

**Figure 6:**
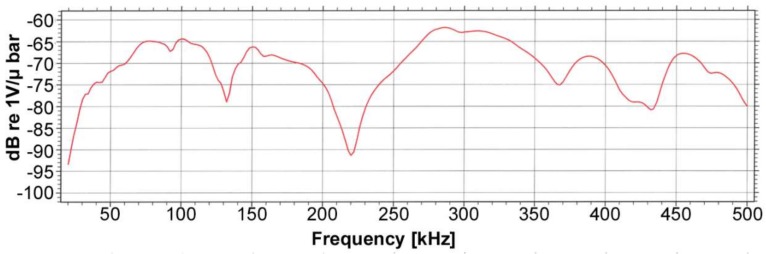
Sensitivity curve of the multi-resonant AE sensors VS45-H.

**Figure 7:**
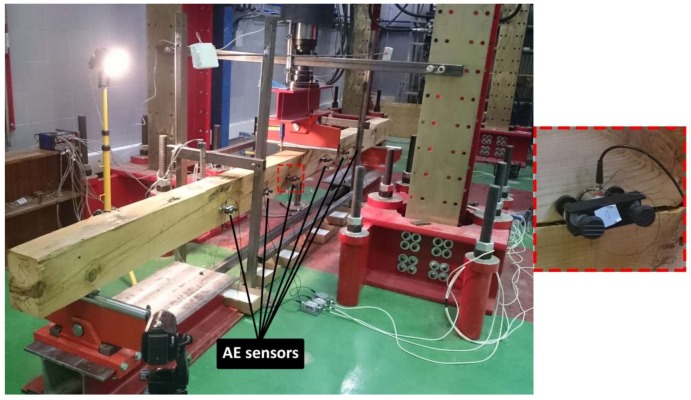
Picture of the BB-NR-1 specimen with the AE sensors.

The large beams were instrumented with 6 AE sensors placed as shown in Figure 5. The position of the AE sensors was established by means of a study of the wave attenuation along specimens. This study was carried out individually for each beam. From this study, the maximum distance between sensors was determined, and then the position of the sensors in the bending tests was established. For reasons of brevity, the results of this study are not included. The AE signals were acquired with Vallen Systeme AMSY-5 equipment, by multi-resonant VS45-H sensors, with sensitivity within the frequency band 20–450 kHz. For waveform recording, the sampling frequency was set at 5 MHz, the number of samples was set at 4096 (i.e., one sample per 0.2 μs) and the pre-trigger was established at 500 samples. For each channel, the threshold was set at 40 dB, and a 34-dB gain preamplifier was used. In order to reject undesirable noises, digital filters in the frequency range 40–500 kHz were used during acquisition. To corroborate the absence of spurious or undesirable signals (some of them produced by the triboelectric effect), a measurement of background noise was carried before each test, i.e., without any load applied to the specimen. In addition, the existence of a low-pass filter at 500 kHz during the acquisition helps with the filtering of possible electromagnetic signals above this frequency. The AE sensors were fixed with magnetic holders, and silicone grease was used for acoustic coupling. Figure 6 and Figure 7 show the sensitivity curve of the AE multi-resonant sensors and a picture of the sensor place on the specimen.

## 4. Results

### 4.1. Mechanical Behavior of Small Beams

**Figure 8:**
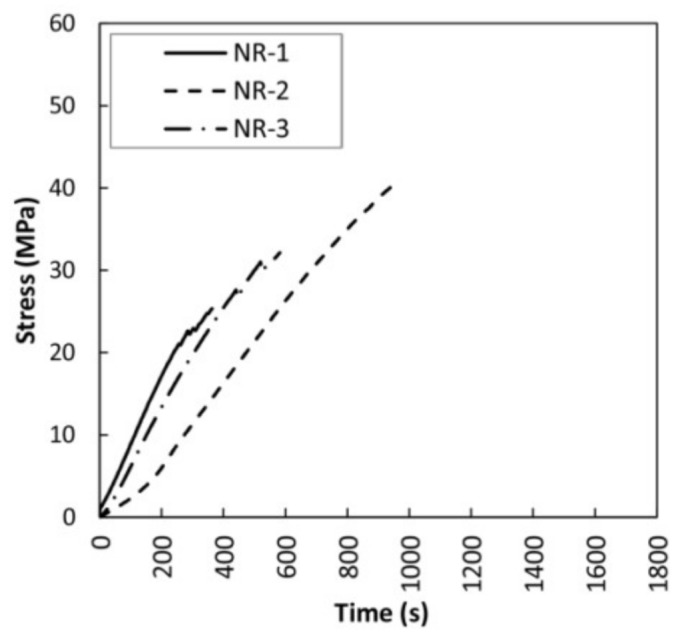
Bending stress versus time for non-reinforced beams (SB-NR).

The bending stress against time for non-reinforced beams (SB-NR) is shown in Figure 8. Table 2 includes the density, maximum bending capacity (MOR), corrected MOR, bending elastic modulus (MOE), maximum deflection and type of failure (classified according to de la Rosa et al. [28]) for each SB-NR specimen. Corrected MOR, defined as in Rescalvo et al. [8] is included to make a fair comparison between all the specimens. This is a usual practice for wood specimens with large number of natural defects.

A clear dispersion in results of the three beams is observed, the main cause being the existence of defects and the different densities of specimens. The failure of all the beams was basically brittle, with crack initiation near the knots. 

**Figure 9:**
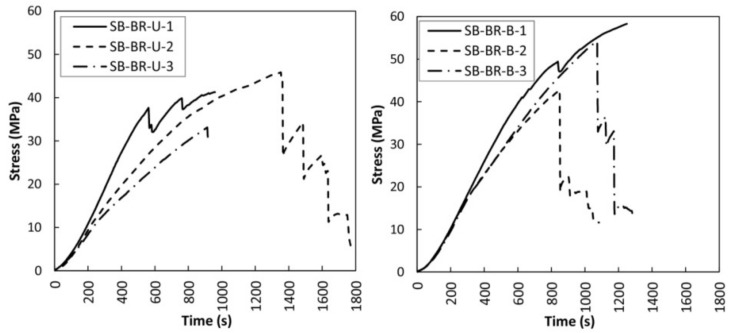
Bending stress versus time for braided reinforced beams (SB-BR). Left: SB-BR-U. Right: SB-BR-B.

The mechanical results obtained for the braided reinforced beams (SB-BR) are shown in Figure 9 and Table 3. A very relevant improvement of the bending stress capacity is achieved for all the cases with respect to the non-reinforced beams. The mean corrected MOR values were 44.99% and 67.63%—higher than for the non-reinforced beams—respectively, for unidirectional and bidirectional fabric. MOE improvement came to 58.81% and 33.46%, while maximum deflection improvements were of 52.90% and 56.64%, respectively. Moreover, a good fastening of the CFRP lamella was achieved with by the discontinuous fabric, which confers a better ductile behavior, as shown in Figure 9.

**Figure 10:**
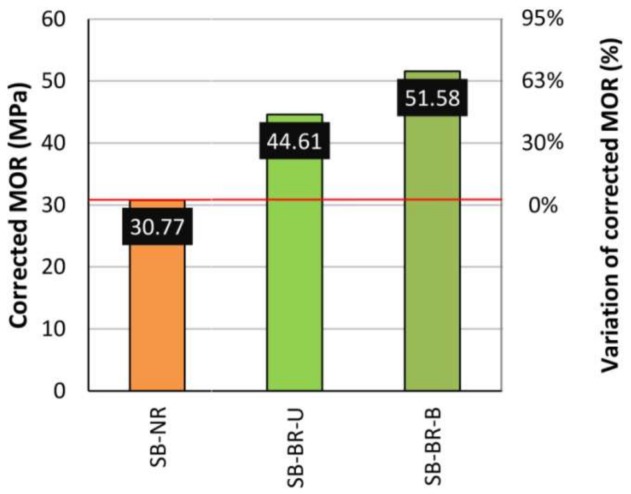
Mean corrected MOR for each beam group. Red horizontal line: Average corrected MOR for SB-NR beams.

As a summary, Figure 10 compares the average corrected MOR for the studied cases and its variation respect to the non-reinforced bemas. Improvements are of about 45% for unidirectional and 65% for bidirectional fabric. It is further evident that, even when there is a high dispersion of density and defect distribution among all the wooden elements, very similar results are obtained for the mean corrected MOR for braided reinforcement layouts. This comes to confirm the advantages of the proposed braided layout, as opposed to the other traditional layouts—in addition to better ductility and the savings in fabric material.

### 4.2. Mechanical Behavior of Large Beams

**Figure 11:**
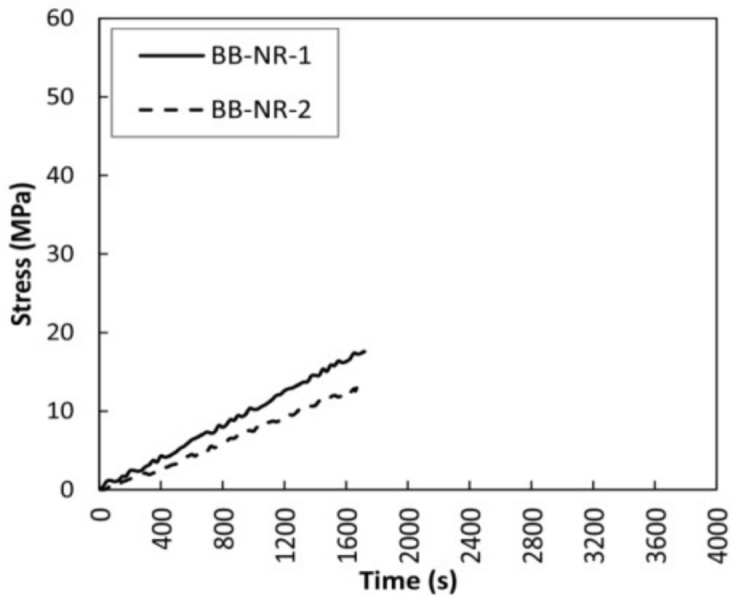
Bending stress versus time for non-reinforced beams (BB-NR).

**Figure 12:**
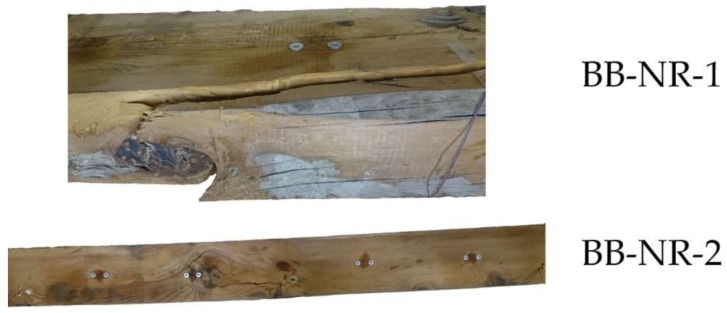
Failure patterns of the BB-NR-1 and BB-NR-2 beams.

Two non-reinforced beams were also used as control specimens, and the mechanical results are shown in Figure 11 and Table 4. The behavior was essentially the same for both beams, with a brittle final failure and a mean corrected MOR of 15.76 MPa, mainly caused by the existence of important knots at the center of the beams. Figure 12 shows the failure patterns of the both beams.

**Figure 13:**
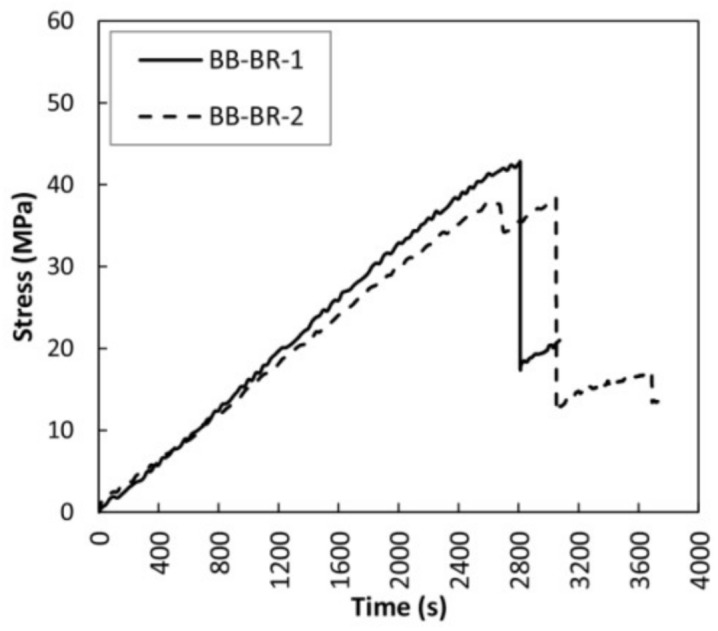
Bending stress versus time for braided reinforced beams (BB-BR).

**Figure 14:**
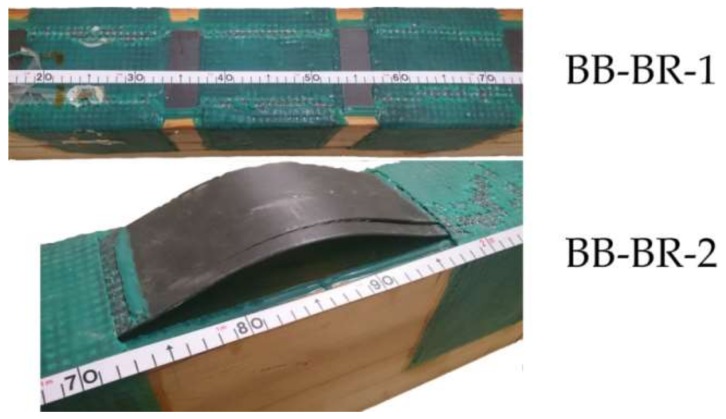
Failure patterns of the BB-BR-1 and BB-BR-2 beams.

The bending stress over time and the mechanical results for the large braided reinforced beams are shown in Figure 13 and Table 5. Bidirectional fabric was chosen as reinforcement, due to having better results than unidirectional fabric in the case of small-sized beams. A very significant improvement of all mechanical properties with regard to the non-reinforced beams is clearly achieved. It is noteworthy that after reaching the maximum bending stress, the beams did not completely exhaust their strength capacity, remaining at 40% and 33% of the maximum value reached for BB-BR-1 and BB-BR-2 beams, respectively. After that point, the bending stress capacity increased again until a particular stress level, with no collapse of stress observed. In other words, a clear improvement in ductility is achieved by means of the braided reinforced layout. The corrected MOR improvement, MOE and maximum deflection was of 163.24%, 98.36% and 69.10% on average, respectively. Figure 14 shows the failure pattern for the two specimens, produced by a final slipping of the CFRP lamella followed by sudden delamination at the areas not covered by the CFRP fabric.

### 4.3. Sensors Monitoring Results

#### 4.3.1. Strain Sensors Data

**Figure 15:**
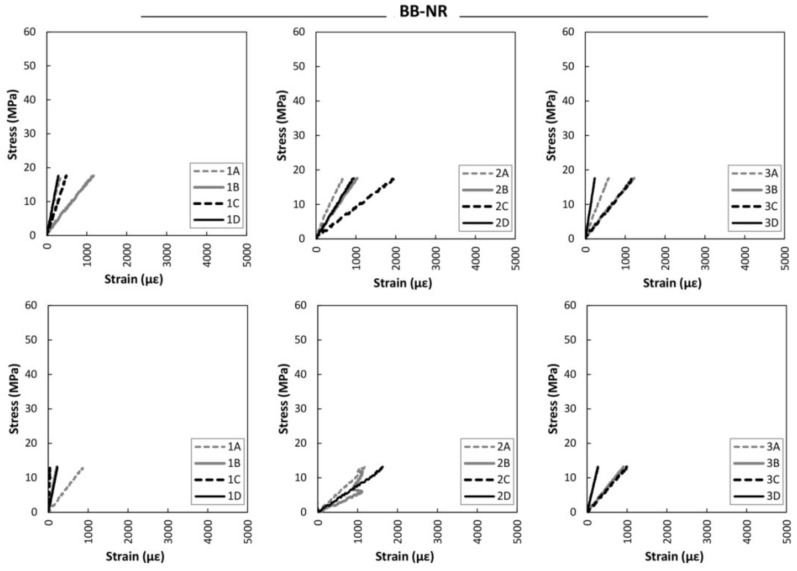
Stress versus strain during bending tests. Top: BB-NR-1. Bottom: BB-NR-2. Left: Strain group 1. Center: Strain group 2. Right: Strain group 3.

**Figure 16:**
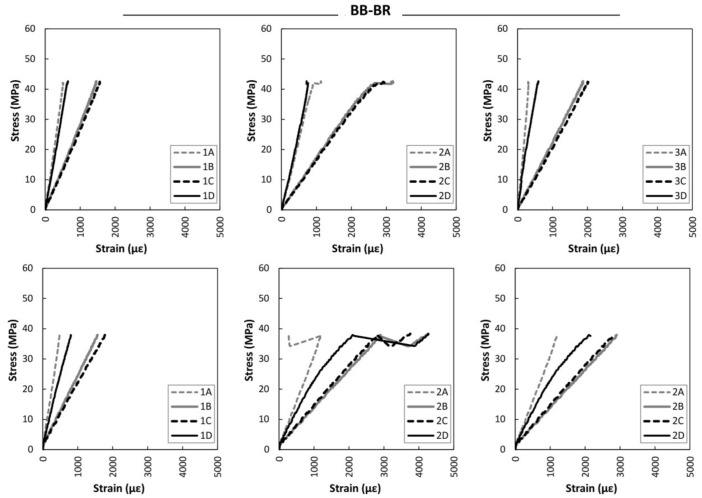
Stress versus strain during bending tests. Top: BB-BR-1. Bottom: BB-BR-2. Left: Strain group 1. Center: Strain group 2. Right: Strain group 3.

Figure 15 gives the strain analysis results obtained for the strain sensors data for the large non-reinforced beams. It is clear that both beams followed a very linear behavior until final breakage, with no plastification in the compression zone. Figure 16 displays the strain analysis up to the maximum bending stress for the large braided reinforced beams. A clear improvement in stiffness, stability and maximum strain is observed, when compared to the non-reinforced beams. Plastification at the compression zone and at the strain gauges of group 2 was also observed during the bending tests.

#### 4.3.2. AE Sensors Results

This sub-section shows the results of the acoustic emission data analysis by using the elastic waves recorded by the AE sensors. For reasons of brevity, only results for BB-NR-1 and BB-BR-2 beams are presented. Due to the fact that the moisture content significantly influences on the wave propagation in the wood, it was measured for all beams during the bending tests. In particular, for the BB-NR-1 and BB-BR-D-2 beams the moisture content was 6.9% and 6.8%, respectively.

**Figure 17:**
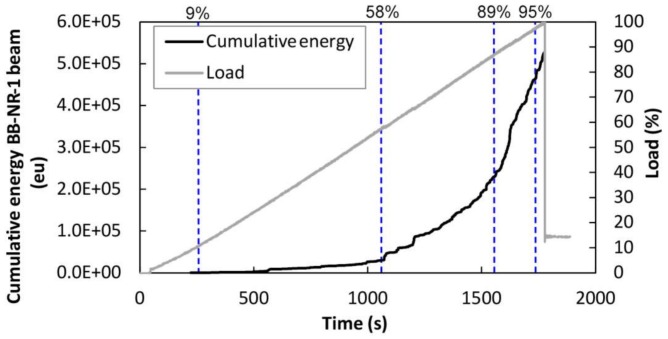
Black line: Cumulative energy versus time. Gray line: Percentage of applied load versus time. BB-NR-1 specimen.

For the case of the BB-NR-1 beam, Figure 17 shows the cumulative AE energy of the events recorded during the bending test over time. The percentage of the applied load is superimposed. During the initial period of the test (0–9% of the load), no AE signals were recorded. This load corresponds to approximately 200 kg, which corresponds to the maximum load supported by the specimen during its life in service, according to the Kaiser effect [13,14]. From this load onward, the accumulative AE energy began to increase, demonstrating the increased damage of the beam. It is widely known that a change in the cumulative energy slope is associated with an important release of strain energy of a given element, due to changes in its stress field produced by new crack formation or the evolution of existing ones. Important increases are observed at about 58% and 89% of the breaking load, which demonstrates that the AE technique gives precursors of the final failure. Dashed lines indicate the percentage of load where changes in the cumulative AE energy curve happened.

**Figure 18:**
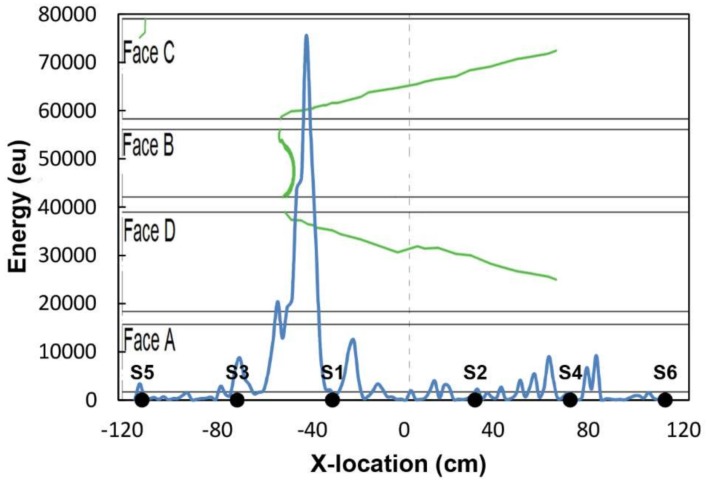
AE location results (AE energy) with respect to the neutral axis of the BB-NR-1 beam at the end of the test. Green line: Crack formation during the bending test (visual inspection). Distances in cm.

Since the tested beams are mainly linear elements (length/height ratio > 20), a linear localization is a reasonable approach that provides enough information for structural conditioning assessment. Accurate 3D location in this kind of materials is a challenging issue that requires a large number of sensors, making monitoring complicated and expensive from an experimental point of view. The propagation wave velocity, obtained for each particular beam by means Hsu-Nielsen pencil lead breaks (2H—0.5mm) made at different points along the sensor line, was 471.17 and 375.21 cm/ms for the BB-NR-1 and BB-BR-2 beams, respectively. Figure 18 shows the location results at the end of the test. Cracks observed at the four faces of the element by visual inspection are represented in green in that Figure, for comparative purposes. The damage pattern basically consisted of a big crack extending from −49 cm (from faces C and D) to 80 cm, approximately. This major crack was also visible from face B. At this point (−49 cm) a large knot was located, which was the cause of the crack, due to the stress concentration at this area. A high concentration of AE energy at the portion (−60, −30) cm, with the maximum peak at −47 cm, is clearly observed, precisely matching the position of the large knot generating the crack. 

**Figure 19:**
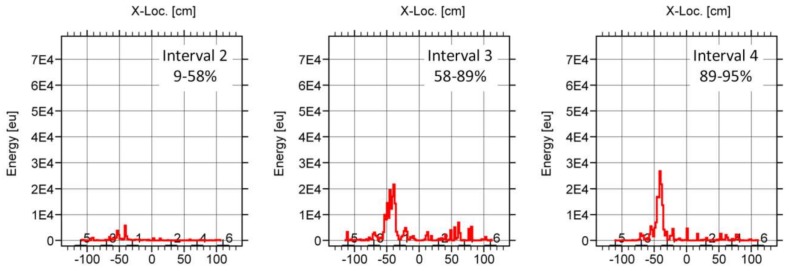
AE location results (AE energy) with respect to the neutral axis of the BB-NR-1 beam of the events recorded at three different intervals of the load.

Finally, the three subfigures of Figure 19 show the location results of the events emitted during three intervals of the loading process. It is remarkable that the AE energy was located the area of the final breakage from the very beginning of the test, demonstrating the potential of the AE technique to predict the weak areas of the beam. A clear increasing of AE energy was reported at both 58–89% and 89–95%, demonstrating the closing to the final breakage of the element. 

**Figure 20:**
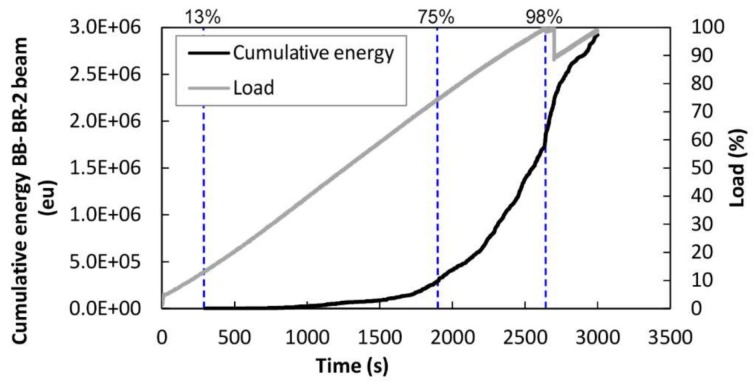
Black line: Cumulative AE energy versus time. Gray line: Percentage of applied load versus time. BB-BR-2 specimen.

For the BB-BR-2 beam, Figure 20 depicts the cumulative AE energy of the events. The load history is also displayed. In this case, up to 13% of the load, no AE signal was recorded, corresponding with approximately 430 kg. From this load onwards, the cumulative AE energy begins to increase, meaning again that the damage increases. Significant increases were observed at 13%, 75% and 98% of the maximum applied load. Dashed lines indicate the changes in the cumulative AE energy curve and the corresponding load percentage.

**Figure 21:**
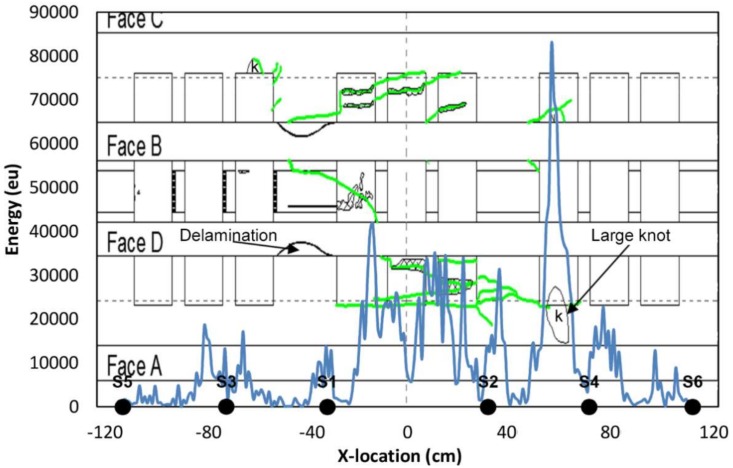
AE location results (AE energy) with respect to the neutral axis of the BB-BR-2 beam at the end of the test. Green line: Crack formation during the bending test (visual inspection). Distances in cm.

**Figure 22:**
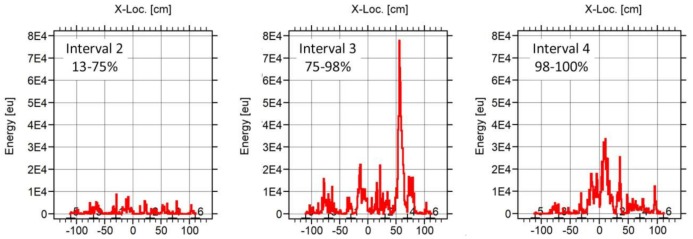
AE location results (AE energy) with respect to the neutral axis of the BB-BR-2 beam of the events recorded at three different intervals of the load.

Figure 21 shows the location results at the end of the test. Cracks on all 4 faces were visually classified, represented in the figure in green colour. In this case, the crack pattern is totally different from the non-reinforced beam, with cracks more distributed along the beam, due to the uniform distribution of the carbon reinforcement. A good match between crack visualization and AE location is observed. The highest concentration of cracks, which probably preceded the failure, is found in the (−30, 30) cm range, i.e., in the middle part of the specimen. Subsequently, the final failure was caused by a partial delamination of around −30 cm (see Figure 14). In addition, a high AE energy concentration is observed around 60 cm. At that point, there was a large knot in the original specimen, with a lot of fissures progressing along the test around it. The breakage of the fibers around the knots releases a high amount of acoustic energy, as they are short fibers with a higher stiffness. Finally, the location results are presented in 3 load ranges (see Figure 22). It can be seen that the large knot zone was active from the beginning of the test, but then stopped, finally being the central area, (−30, 30) cm where the wood-CFRP delamination was observed, the final failure zone, in wood agreement with final observation.

#### 4.3.3. Comparison of AE and Strain Sensors Measurements

With the aim of comparing the AE measurements with those registered by the strain sensors, a SR (Strain Ratio) coefficient is proposed as follows:

(1) $$SR_{Xb,Yl}=\frac{\varepsilon_{b}}{\varepsilon_{l}}$$

where *ε_b_* is the strain registered by a strain sensor placed on the bottom face (sensors B or C) and *ε_l_* is the strain recorded by lateral strain sensor (A or D). As mentioned in Rescalvo et al. [27], this relationship also provides real information about the position of the neutral fiber. (*X_b_*,*Y_l_*) represents the name of the used sensors to calculate SR.

**Figure 23:**
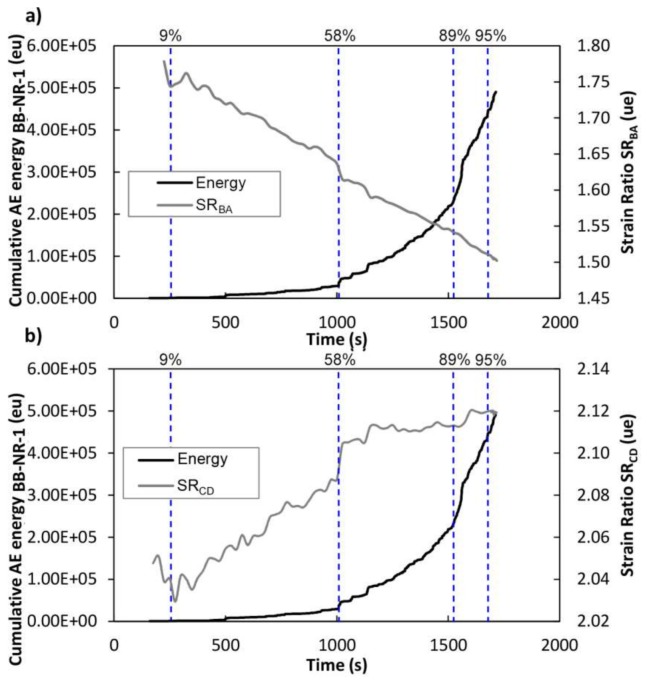
Beam BB-NR-1. Black line: Cumulative AE energy versus time. Gray line: Strain Ratio versus Time, (**a**) Strain Ratio recorded by strain sensor A and B, (**b**) Strain Ratio recorded by strain sensor C and D. Dashed line: Percentage of load.

**Figure 24:**
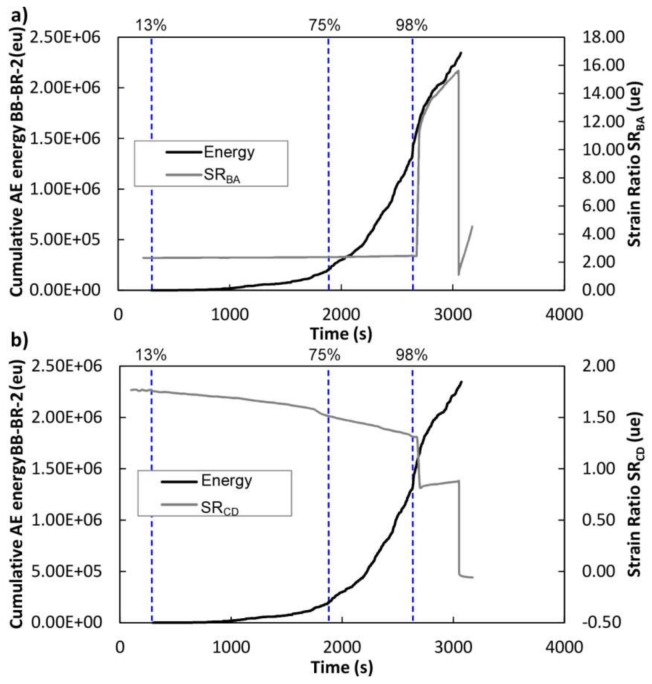
Beam BB-BR-2. Black line: Cumulative AE energy versus time. Gray line: Strain Ratio versus Time, (**a**) Strain Ratio recorded by strain sensor A and B, (**b**) Strain Ratio recorded by strain sensor C and D. Dashed line: Percentage of load.

In particular, Figure 23 and Figure 24 show the SR_BA_ and SR_CD_ for the mid-span strain sensor group for BB-NR-1 and BB-BR-2 beams, respectively, superimposed on the cumulative AE energy. In order to make a reliable comparison, dashed vertical lines indicate the changes in the cumulative AE energy curve described above, with the corresponding load percentage.

Figure 23 and Figure 24 demonstrate that, in general, changes in SR and the AE-energy slope are correlated. In particular, for the beam BB-NR-1, at 58% of the load both the AE energy and the SR parameter have significant changes in their slopes. For the 89% of the load, the AE-energy slope changes, also corresponding with a variation in the SR_CD_ parameter. This change is not well observed in the SR_BA_ parameter, probably due to heterogeneity of wood and the non-uniform distribution of the damage. At 89% of the load, a slight increase of SR_CD_ is also observed. 

For the case of the reinforced beam BB-BR-2, at 75% of the load, only the SR_CD_ parameter slightly changes. However, at 98%, close to the maximum load, both strain ratios show a clear and remarkable change, in good match with the increase of the AE results.

## 5. Conclusions

The acoustic emission data recorded by using multi-resonant piezoelectric sensors demonstrated how this information serves for real-time monitoring of the wood beam elements. Most notably:

- The increasing level of damage in the element induces very significant increases in the released AE, a key point when considering structural safety.
- The linear location results match the visual observation of the damage very closely. More specifically, AE located the stress concentration produced by knots in the wood and final existence of CFRP-wood delamination. The AE linear location confirmed that AE can announce well in advance the location of a main damage area, which is very important for future interventions entailing the repair or conservation of wood beams.
- A good correlation exists between measurements of AE and strain sensors, thus confirming the validity of the AE methodology for SHM of this kind of structures.

The AE monitoring process has been carried out on non-reinforced and reinforced beams. In particular, a layout was proposed by our research group for the reinforcement of timber beams by using carbon composite materials, tentatively presented as braided reinforcement. The layout essentially consists of a combination of CFRP lamella attached on the tension side and the use of CFRP fabric discontinuously wrapping the timber element. The experimental program involved old timber beams of pine, with more than 200 years in service, comparing unidirectional and bidirectional fabrics. Bending tests were performed on small-sized beams (1.3 m long). Verification of the improvement over non-reinforced beams was also carried out on large (4.5 m long) beams. The main conclusions are:

- Although both unidirectional and bidirectional fabrics provided relevant improvements in bending load capacity, the bidirectional ones demonstrated a 15.5% better performance on average than the unidirectional ones.
- Bending tests on large beams provided an improvement of 163% in terms of the corrected MOR and 90% in stiffness, in comparison with the non-reinforced beams, confirming the advantages of Braided Reinforcement in situations close to the field applications.

It is important to highlight that for the application of the reinforcement, it is totally necessary to have good control of the internal moisture content of wood, as well as its variation. This aspect is especially critical during the drying period of the epoxy resin, as it will not acquire all its mechanical properties until this period is completed. Therefore, during this phase, volumetric changes due to moisture variation could seriously affect the wood-CFRP bond.

While the validity of the real-time AE monitoring has been demonstrated in this paper, works such as [29] use waveform parameters to characterize the fracture process and the delamination of the CFRP. The use of these parameters, although not trivial (especially in the case of old wood with large heterogeneities), is of great interest, reason why it is currently under analysis in our research group.

## Figures and Tables

**Table 1:** Experimental program for bending tests: specimens and nomenclature.

Size of Timber Beam	Reinforcement Layout	Width of the CFRP Lamella (mm)	CFRP Fabric	Number of Beams	Short Name
Small-sized beams (SB)	Non- Reinforced (NR)	-	-	3	SB-NR
	Braided Reinforcement (BR)	45	Laminate + Unidirectional wrap (U)	3	SB-BR-U
		45	Laminate + Bidirectional wrap (B)	3	SB-BR-B
Large-sized beams (BB)	Non- Reinforced (NR)	-	-	2	BB-NR
	Braided Reinforcement (BR)	100	Laminate + Bidirectional wrap (B)	2	BB-BR-B

**Table 2:** Mechanical results of non-reinforced beams (SB-NR).

Name	MOR (MPa)	Density (kg/m^3^)	Corrected MOR (MPa)	MOE (MPa)	Maximum Deflection (mm)	Type of Failure
SB-NR-1	25.74	692.17	22.10	13,410.55	10.39	Tension
SB-NR-2	43.63	653.77	39.66	14,978.33	18.52	Tension and shear
SB-NR-3	32.88	639.57	30.55	7508.31	15.21	Shear
Mean SB-NR value	34.08	661.84	30.77	11,965.73	14.71	-

**Table 3:** Mechanical results for braided reinforced beams (SB-BR).

Name	MOR (MPa)	Density (kg/m^3^)	Corrected MOR (MPa)	MOE (MPa)	Maximum Deflection (mm)	Type of Failure
SB-BR-U-1	41.28	688.30	35.64	16,870.84	22.71	Shear
SB-BR-U-2	45.83	521.17	52.26	18,491.64	24.79	Tension and shear
SB-BR-U-3	33.14	428.69	45.94	18,647.46	19.96	Tension and shear
Mean SB-BR-U value	40.09	546.05	44.61	18,003.31	22.49	-
Variation respect NR (%)	17.61	-	44.99	58.81	52.90	-
SB-BR-B-1	58.31	549.73	63.03	13,439.87	27.30	Shear
SB-BR-B-2	42.86	628.10	40.55	17,591.69	19.15	Shear
SB-BR-B-3	54.08	628.26	51.15	16,875.22	22.66	Tension and shear
Mean SB-BR-B value	51.75	602.03	51.58	15,968.93	23.04	-
Variation respect NR (%)	51.83	−9.04	67.63	33.46	56.64	-

**Table 4:** Mechanical results from bending tests for large non-reinforced beams (BB-NR).

Name	MOR (MPa)	Density (kg/m^3^)	Corrected MOR (MPa)	MOE (MPa)	Maximum Deflection (mm)	Type of Failure
BB-NR-1	17.61	522.52	18.71	9239.25	34.86	Tension and shear
BB-NR-2	13.19	571.12	12.82	6384.22	35.43	Tension
Mean BB-NR value	15.40	546.82	15.76	7811.74	35.15	-

**Table 5:** Mechanical results from bending tests for braided reinforced beams (BB-BR).

Name	MOR (MPa)	Density (kg/m^3^)	Corrected MOR (MPa)	MOE (MPa)	Maximum Deflection (mm)	Type of Failure
BB-BR-1	42.88	468.72	50.78	16,858.18	59.95	Tension and shear
BB-BR-2	38.19	658.11	32.21	14,131.79	58.91	Tension and shear
Mean BB-BR value	40.54	563.42	41.50	15,494.99	59.43	-
Variation respect BB-NR (%)	163.21	-	163.24	98.36	69.10	-

## References

[1] Commission E. (2010). Directive 2010/31/EU of the European parliament and of the council of 19 May 2010 on the energy performance of buildings. Off. J. Eur. Commun..

[2] Ansell M.P. (2015). Hybrid wood composites-integration of wood with other engineering materials. Wood Composites.

[3] Argüelles R., Arriaga F., Martínez J.J. (1996). Estructuras de Madera, Diseño y Cálculo.

[4] High performance Synthetic Composites; Manufacturing, Recent Developments and Applications. https://textiletoday.com.bd/high-performance-synthetic-composites-manufacturing-recent-developments-and-applications/.

[5] Theakston F. (1965). A feasibility study for strengthening timber beams with fiberglass. Can. Agric. Eng..

[6] Biblis E. (1965). Analysis of wood-fiberglass composite beams within and beyond the elastic region. FPJ.

[7] Kellogg R., Wangaard F. (1964). Influence of fiber strength on sheet properties of hardwood pulps. Tappi.

[8] Rescalvo F.J., Valverde-Palacios I., Suarez E., Gallego A. (2017). Experimental comparison of different carbon fiber composites in reinforcement layouts for wooden beams of historical buildings. Materials.

[9] Chen B., Liu J. (2008). Damage in carbon fiber-reinforced concrete, monitored by both electrical resistance measurement and acoustic emission analysis. Constr. Build. Mater..

[10] Niccolini G., Borla O., Accornero F., Lacidogna G., Carpinteri A. (2015). Scaling in damage by electrical resistance measurements: An application to the terracotta statues of the sacred mountain of varallo renaissance complex (Italy). Rendiconti Lincei.

[11] Grosse C.U., Ohtsu M. (2008). Acoustic Emission Testing.

[12] Ono K. (2011). Application of acoustic emission for structure diagnosis. Diagnostyka.

[13] Mizutani Y. (2016). Practical Acoustic Emission Testing (The Japanese Society for Non-Destructive Inspection).

[14] Gallego A., Martínez E. (2016). Emisión acústica. Niveles I y II (Ensayos no Destructivos-AEND).

[15] Sause M.G. (2016). In Situ Monitoring of Fiber-Reinforced Composites: Theory, Basic Concepts, Methods, and Applications.

[16] Sagasta F., Benavent-Climent A., Roldán A., Gallego A. (2016). Correlation of plastic strain energy and acoustic emission energy in reinforced concrete structures. Appl. Sci..

[17] Miller R.K., Hill E.V.K., Moore P.O. (2005). Acoustic Emission Testing (AE), Nondestructive Testing Handbook.

[18] Rescalvo F.J., Valverde-Palacios I., Suárez E., Gallego A. Monitoring of debondings in timber beams reinforced with using acoustic emission technique. Proceedings of the 8th International Conference on Acoustic Emission (IIIAE 2016).

[19] Ono K., Gallego A. Research and applications of AE on advanced composites. Proceedings of the 30th European Conference on Acoustic Emission Testing.

[20] Martínez-Jequier J., Gallego A., Suárez E., Juanes F.J., Valea Á. (2015). Real-time damage mechanisms assessment in CFRP samples via acoustic emission lamb wave modal analysis. Compos. Part B-Eng..

[21] Kowalski S.J., Moliński W., Musielak G. (2004). The identification of fracture in dried wood based on theoretical modelling and acoustic emission. Wood Sci. Technol..

[22] Gozdecki C., Smardzewski J. (2005). Detection of failures of adhesively bonded joints using the acoustic emission method. Holzforschung.

[23] Ritschel F., Brunner A.J., Niemz P. (2013). Nondestructive evaluation of damage accumulation in tensile test specimens made from solid wood and layered wood materials. Compos. Struct..

[24] Lamy F., Takarli M., Angellier N., Dubois F., Pop O. (2015). Acoustic emission technique for fracture analysis in wood materials. Int. J. Fract..

[25] Diakhaté M., Bastidas-Arteaga E., Pitti R.M., Schoefs F. (2017). Cluster analysis of acoustic emission activity within wood material: Towards a real-time monitoring of crack tip propagation. Eng. Fract. Mech..

[26] Diakhate M., Angellier N., Pitti R.M., Dubois F. (2017). On the crack tip propagation monitoring within wood material: Cluster analysis of acoustic emission data compared with numerical modelling. Constr. Build. Mater..

[27] Rescalvo F.J., Valverde-Palacios I., Suarez E., Gallego A. (2018). Experimental and analytical analysis for bending load capacity of old timber beams with defects when reinforced with carbon fiber strips. Compos. Struct..

[28] De la Rosa García P., Escamilla A.C., García M.N.G. (2013). Bending reinforcement of timber beams with composite carbon fiber and basalt fiber materials. Compos. Part B-Eng..

[29] Aggelis D.G., Verbruggen S., Tsangouri E., Tysmans T., Van Hemelrijck D. (2013). Characterization of mechanical performance of concrete beams with external reinforcement by acoustic emission and digital image correlation. Constr. Build. Mater..

